# Resistance training restores skeletal muscle atrophy and satellite cell content in an animal model of Alzheimer’s disease

**DOI:** 10.1038/s41598-023-29406-1

**Published:** 2023-02-13

**Authors:** Masoud Rahmati, Mohammad Shariatzadeh Joneydi, Ai Koyanagi, Guang Yang, Bingzhou Ji, Seung Won Lee, Dong Keon Yon, Lee Smith, Jae Il Shin, Yusheng Li

**Affiliations:** 1 Department of Physical Education and Sport Sciences, Faculty of Literature and Human Sciences, Lorestan University, Khorramabad, Iran; 2 Sport Sciences Research Institute of Iran, Tehran, Iran; 3 Parc Sanitari Sant Joan de Deu/CIBERSAM, ISCIII, Universitat de Barcelona, Fundacio Sant Joan de Deu, Sant Boi de Llobregat, Barcelona, Spain; 4 ICREA (Catalan Institution for Research and Advanced Studies), Barcelona, Spain; 5 Deparment of Orthopedics, Xiangya Hospital, Central South University, Changsha, Hunan China; 6 Sungkyunkwan University School of Medicine, Suwon, Republic of Korea; 7 Medical Science Research Institute, Kyung Hee University College of Medicine, Seoul, Republic of Korea; 8 Department of Pediatrics, Kyung Hee University Medical Center, Kyung Hee University College of Medicine, Seoul, Republic of Korea; 9 Centre for Health, Performance, and Wellbeing, Anglia Ruskin University, Cambridge, UK; 10 Department of Pediatrics, Yonsei University College of Medicine, Seoul, Republic of Korea

**Keywords:** Stem cells, Health care

## Abstract

Alzheimer’s disease (AD) is the most common neurodegenerative disease, and numerous recent findings suggest that several pathologic signs, including loss of muscle strength and mass, are also detected in these patients. In the present study, we evaluated muscle cross-sectional area (CSA), myonuclear number, satellite cell (SC) content, and myosin heavy chain (MyHC) types in an animal model of AD and examined the possible role of resistance training in controlling skeletal muscle size in this disease. Fifty-eight male rats were randomly divided into four groups: healthy-control (H-C), healthy-exercise (H-Ex), Alzheimer-control (A-C), and Alzheimer-exercise (A-Ex). AD was induced by the single injection of 1–42 amyloid into the CA1 region of the hippocampus (1 μl/site). The rats in H-Ex and A-Ex groups performed a 5-week resistance training period (17 sessions). The results indicated that AD induces significant skeletal muscle atrophy and reduces the myonuclear number and SC content in *gastrocnemius* muscle in both whole muscle cross-sections and isolated myofibers. Interestingly, we did not find any significant differences in the different MyHC distributions of AD animals compared with controls, while resistance training significantly increased the CSA of MyHC IIb fibers in both AD and healthy animals. Altogether, these observations suggest that the skeletal muscle of AD animals are more prone to atrophy and loss of myonuclear number and satellite cell content, while resistance training successfully restores these impairments.

## Introduction

Alzheimer’s disease (AD) is the most common form of cognitive impairment and neurodegenerative disease affecting nearly 10% of individuals over 65 and 50% of those over 85^1^. The brain of AD patients is characterized by the deposition of amyloid plaques and accelerated atrophy in the brain’s gray matter cortex, reflecting the loss of neurons in areas such as the hippocampus and parietal lobes^2^. Moreover, in addition to neurodegenerative features and cognitive impairments, patients suffering from AD often exhibit reduced skeletal muscle mass and strength. Such deficits have been considered an early sign of AD, which may help predict the onset of later AD development^3^. Furthermore, skeletal muscle deficits contribute to disability and loss of functional independence in AD patients^4^. Although there is no information about the exact underlying mechanism involved in such skeletal muscle deficits in AD patients, a testable hypothesis is that common pathological processes co-occur in the brain and skeletal muscle^3,4^. In this regard, abnormal amyloid precursor protein (APP) metabolism with amyloid-β deposition has been detected in skeletal muscle tissue of AD patients^5^. Moreover, abnormal body weight loss and cachexia are clinical criteria for diagnosing AD^6^. Experimental data indicated that progressive brain atrophy in AD patients positively correlates with reduced muscle mass, suggesting that pathological processes may co-exist in the brain and skeletal muscle^4^.

The skeletal muscle is a dynamic and highly plastic tissue capable of remodeling its properties in response to various stimuli such as chronic diseases and exercise training^7,8^. Satellite cells (SCs), the skeletal muscle stem cell population, are the primary contributors to the maintenance and repair of skeletal muscle and play a central role in skeletal muscle plasticity^9^. Studies have shown that resistance training-induced hypertrophy depends on the SC pool in human skeletal muscle^10,11^. Although SCs are fundamentally involved in maintaining the size of the skeletal muscle, no study has investigated SC populations in Alzheimer’s disease. The current study aimed to assess SC content in an animal model of Alzheimer’s disease and to examine the possible role of resistance training in controlling skeletal muscle size in this disease. We hypothesise that skeletal muscle tissue in an animal model of Alzheimer’s disease possesses atrophy and reduced SC content and that resistance training will prevent from these deleterious effects.

## Materials and methods

### Animals and muscle tissue preparation

All experiments involving animals were performed following approved guidelines and ethical approval from Sport Sciences Research’s Institutional Animal Care and Use Committee (as registered under the code: IR.SSRI.REC.1401.1732) and according to the NIH Guidelines for the Care and Use of Laboratory Animals (NIH publication, 1996). Additionally, the present study was carried out in compliance with the ARRIVE guidelines. Two-month-old Wistar male rats (n = 48) were purchased from Lorestan University of Medical Sciences Laboratories and housed three-per-cage in an animal lab under standard conditions (12-h light/dark cycle in a room at a temperature of 20–25 °C) with access to food and water ad libitum. The animals were randomly assigned into four equal groups of 12: healthy-control (H-C), healthy-exercise (H-Ex), Alzheimer-control (A-C), and Alzheimer-exercise (A-Ex). At the end of the treatment periods, all rats were anesthetized with isoflurane inhalation. *Gastrocnemius* muscles from animals in all groups were dissected in optimal cutting temperature (OCT) medium, mounted on pieces of cork, secured with tragacanth gum, and frozen in liquid nitrogen-cooled isopentane and further stored at − 80 °C. Ten µm-thick cryosections were prepared and processed for immunostaining and used to test the program’s ability to recognize myofiber morphology and SCs content.

### Alzheimer’s induction

To induce Alzheimer's disease in the present study, beta-amyloid 1–42 (Aβ_1-42_) was injected into the CA1 region of the dorsal hippocampus of Aβ rats using a stereotaxic device (RWD, China)^12^. To prepare the injection solution, 1 mg/mL of Aβ_1-42_ (Abcam, Cambridge, UK, Cat no. ab120301) was initially diluted with ice-cold 1,1,1,3,3,3-hexafluoro-2-propanol (HFIP) and incubated at room temperature for at least 60 min with occasional vortexing. Next, the HFIP was removed using a SpeedVac, and the peptide film was stored at − 80 °C. Then for aggregation, the peptide was first resuspended in DMSO (5 mM), sterile phosphate buffer saline (PBS) was added to bring the peptide to a final concentration of 1 μg/μL, and the Aβ_1-42_ peptide was incubated at 37 °C for 72 h. To perform stereotaxic surgery, the animals were anesthetized with isoflurane inhalation. Aβ_1-42_ suspension (1 μl/site) was injected into the CA1 region of the dorsal hippocampus of A-C, and A-Ex groups (A-4.2, L ± 3.0, V-2.0 mm) based on the Paxinos and Watson atlas by a Hamilton syringe attached to an infusion pump^12^. Moreover, isotonic saline solution was injected into the sham animals. To confirm Alzheimer’s induction, pathological slides were prepared from the hippocampus of three animals/per group 10 days after surgery using thioflavin immunostaining of beta-amyloid plaques.

### Resistance training protocol

The rats in the H-Ex and A-Ex groups performed a 4-week training period. Resistance training was conducted using a 1-m ladder with 26 rungs and an inclined at 85° with a house chamber (20 × 20 × 20 cm) placed at the top. Initially, the rats were familiarized for one week with climbing ladders, and then the resistance training protocol was implemented for four weeks. Accordingly, in the familiarization period, the animals were taught to climb the ladder and perform four trials per day for four days. The rats were placed at the bottom of the climbing ladder with weights attached to their tails and were motivated to climb by touching the tail with tweezers to initiate the movement. Once the rats climbed to the house chamber they were allowed to rest inside the chamber for 120 s. The familiarization protocol was repeated until the animals voluntarily climbed the ladder three consecutive times without stimuli. Then, resistance training with progressively heavier loads was performed for four weeks at least with 48 h rest between each training session (12 sessions; on the same days each week: Saturday, Monday, and Wednesday). To determine the maximal carrying load (MCL), on the first day of every training week, each animal carried a load that was 75% of their body mass, and 30 g were added for each additional climb repetition until the rat was unable to climb the entire length of the ladder. The next training sessions consisted of five ladder climbs with 65, 75, 85, 95, and 100% of the rat’s previous MCL, as determined on the first day of every training week. Each resistance training session consisted of 5 sets / 4 reps with a 60-s break between reps and 3 min between sets. This type of resistance training protocol was adapted from the previous reports^13–15^, and according to the needs of the current study.

### Morris Water Maze test

To study spatial learning and memory, we used Morris Water Maze (MWM) according to our previous studies^15,16^. In summary, in the fourth week of resistance training, the MWM test was performed to test the animals’ cognition, including spatial learning and memory. MWM consisted of the following apparatus: (1) a black circular pool filled with water (22 °C ± 2 °C, 200 cm diameter, walls 76 cm depth, divided into four quadrants: N, S, W, and E), located in a room with visual signs on the walls equipped with a computerized tracking/image analyzer system, and (2) a platform with a diameter of 10 cm and a height of 35 cm, which was placed 2 cm below the water's surface and in the middle of the S and E quartile during the spatial learning test. To test the animal spatial learning, the rats were randomly abandoned from the N, S, W, and E points in the water. In the spatial learning phase, (four trials/day for four consecutive days), rats were given up to 60 s per trial to find the hidden platform and were required to remain seated on the platform for 10 s, otherwise, the rats were guided by hand and allowed to remain on the platform for 10 s (in this case their escape latency was accepted as 60 s). Then, 24 h after the last spatial learning test, rats were tested for spatial memory (probe trial). To test the probe trial, after removing the platform, the time spent in the target quadrant was recorded for up to 60 s.

### Biochemical assays for SOD, CAT, GPx, GSH, and MDA

All biochemical assays in the present study were carried out according to our previous study^15^. In summary, the activities of Superoxide dismutase (SOD), Catalase activity (CAT), and glutathione peroxidase (GSH) in the homogenate of the *gastrocnemius* muscle were measured by the specific ELISA kits developed for rats according to their manuals instruction (ZellBio GmbH, Germany).

### Immunofluorescent staining

All immunohistochemical procedures in the present study were carried out according to our previous study^7^. To detect different myofibers, *gastrocnemius* muscle Sects. (10 µm-thick) were incubated with antibodies specific to myosin heavy chain (MyHC) types I, IIa, and IIb (BA-D5, SC-71, and BF-F3, respectively, University of Iowa Developmental Studies Hybridoma Bank, Iowa City, IA), supplemented with rabbit polyclonal anti-laminin antibody (L9393; Sigma-Aldrich, St. Louis, MO). Unstained myofibers were judged as MyHC IIx expression. Alexa Fluor 405, 488, and 546 secondary antibodies were used to detect MyHC types I, IIa, and IIb, respectively (Molecular Probes, Thermo Fisher Scientific, Waltham, MA, USA). Further, laminin (L9393 Sigma-Aldrich, St. Louis, MO, USA) and Pax7 (Developmental Studies Hybridoma Bank, Iowa, IA, USA) were used to detect myofiber borders and satellite cells, respectively. Secondary antibodies coupled to anti-rabbit IgG Cy3 and Cy5-labeled (Jackson Immunoresearch Labs, West Grove, PA, USA) were used to detect laminin and Pax7.

### Image acquisition and quantification

All images were captured at × 10 (for cross-sectional area (CSA) and fiber type measurements) and × 20 (for myonuclei and SCs measurements) magnification using a Carl Zeiss AxioImager fluorescent microscope (Carl Zeiss, Jena, Germany). To analyze whole muscle cross-section, consecutive fields from whole muscle sections were automatically acquired in multiple channels using the mosaic function in Image M1 Software (version 4.9.1.0, RRID: SCR_002677). For automatic quantification of mean CSA, myonuclei, fiber-type, and SCs images from various experimental conditions were analyzed using MyoView software as described^7^.

### Imunofluorescence staining and morphometric analysis of isolated myofibers

In order to immunofluorescence staining of mono-myofibers, the *gastrocnemius* muscle blocks were fixed in 4%PFA in PBS for 2H at RT. After several washes, 40 to 50 mono-myofibers were isolated per staining from each muscle. Then, isolated myofibers were mounted on glass slides in Vectashield mounting medium containing DAPI (Vector Laboratories, UK). To detect myonuclei, myofibers were mounted on slides using fluromount Aqueous mounting (Sigma, F4680-25 mL) and kept at 4° C. Isolated myofibers were analyzed using confocal laser scanning microscopy (Olympus Co. Ltd., Tokyo, Japan). CSA of isolated myofibers was calculated by the following formulas: CSA = *π* × (*w*/2) × (*t*/2) where *w* and *t* are the width and thickness, respectively. Additionally, the number of nuclei was counted and illustrated as the number of nuclei/100 µm fiber length or the number of nuclei/volume calculated for 100 µm fiber length. All of these analyses were determined on image stacks using the 3D manager plugin of Image J software^17^.

### Statistical analyses

Statistical analysis was performed using the Graph-Pad Prism statistics software (Graph-Pad Software Inc., San Diego, La Jolla CA, USA free demo version 8.0.0, www.graphpad.com) and data was reported as mean ± S.E.M. Shapiro–Wilk and Levin’s tests were used to check normal data distribution and to determine the homogeneity of variances, respectively. Two-way ANOVA followed by Tukey’s post hoc test was performed for CSA, myofiber types, myonuclei and SC content changes with the resistance training program. Values of *p* < 0.05 were considered statistically significant.

## Results

### Alzheimer’s confirmation

**Figure 1:**
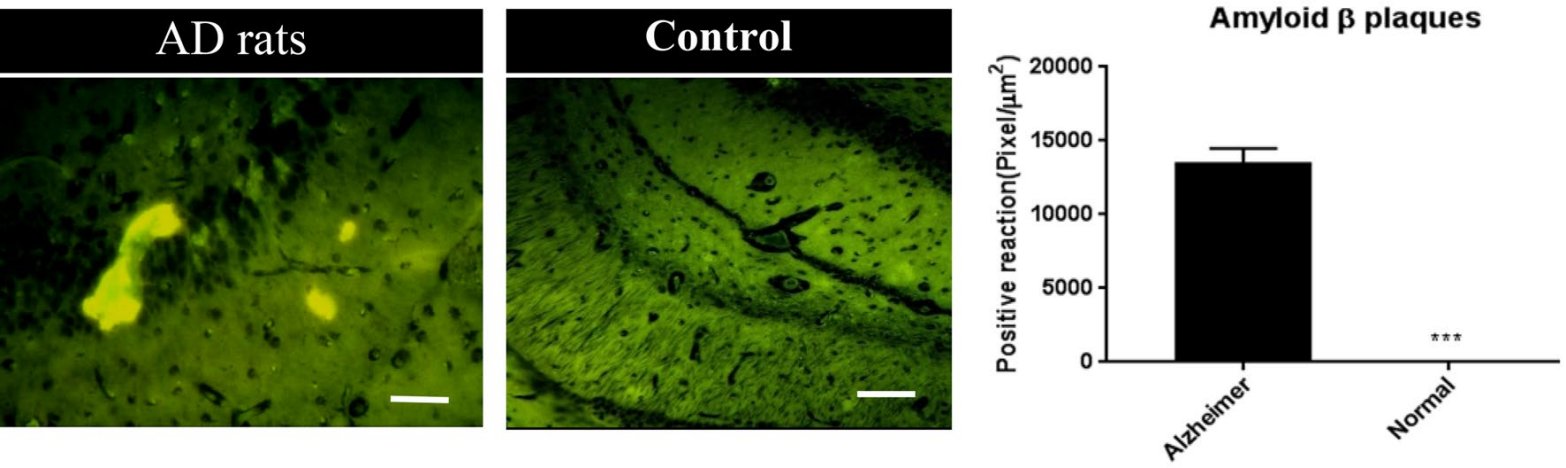
Photomicrographs of thioflavin staining to assess beta-amyloid plaques in the hippocampus of control and Alzheimer’s rats. After confirming Alzheimer's model in animals, the rats were randomly assigned in to two groups (A-C and A-Ex). Scale bar = 50 µm. ***p < 0.0001.

To assess the formation of amyloid plaques in AD rats, 10 days after the induction of Aβ_1-42_, the Alzheimer's model was confirmed using thioflavin staining. The mean expression of beta-amyloid plaques was 29.49 ± 10.74% in the control groups and 14,876 ± 573.4% in the AD groups (*p* = 0.001) (Fig. 1).

### Resistance training improved cognitive impairment in an animal model of Alzheimer’s disease

**Figure 2:**
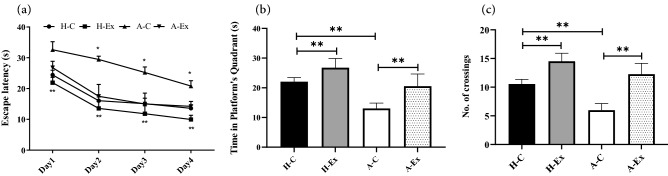
Resistance training improves cognitive impairment in an animal model of Alzheimer’s disease. Results extracted from Morris Water Maze (MWM) test in spatial learning (Morris water maze task) (**a**), spatial memory (probe trial) (**b**), and number of crossing across platform (probe trial) (**c**). In (**a**) *p < 0.001 for A-C group vs. other groups, **p < 0.001 for H-Ex group versus A-Ex group. In (**b**, **c**) **p < 0.001; (Two-way ANOVA and subsequent Tukey’s HSD).

Cognitive dysfunction is a consequence of AD, leading to memory and learning impairments. We examined the effects of AD and resistance training on cognitive function with the MWM test in an animal model of Alzheimer's disease. As shown in Fig. 2a, in the MWM test, significant differences in escape latency of different groups were observed on the 1st, 2nd, 3rd, and 4th days, showing that AD in rats in A-C group showed significantly higher escape latency on the 1st, 2nd, 3rd and 4th days compared to the H-C group rats (all *p* < 0.001). Resistance training in animals in H-Ex and A-Ex groups resulted in improvement in learning between trials from day 1 to 4 and decreased in escape latency compared to the H-C and A-C groups, respectively (all *p* < 0.001). As shown in Fig. 2b, c, significant difference was observed in the time spent in the target quadrant and the number of crossings across the platform area (probe trial) of different experimental groups. The time spent in the target quadrant and the number of crossings were significantly decreased in A-C rats compared to H-C rats (*p* < 0.001). Resistance training in animals in H-Ex and A-Ex groups improved the time spent in the target quadrant, and the number of crossings across the platform area compared to the H-C and A-C groups, respectively (all *p* < 0.001). Taken together, these results implicated that AD could lead to cognitive impairment and resistance training was capable to improve cognitive function in an animal model of AD.

### Resistance training successfully restored Alzheimer’s-induced muscle atrophy and myonuclear reduction

**Figure 3:**
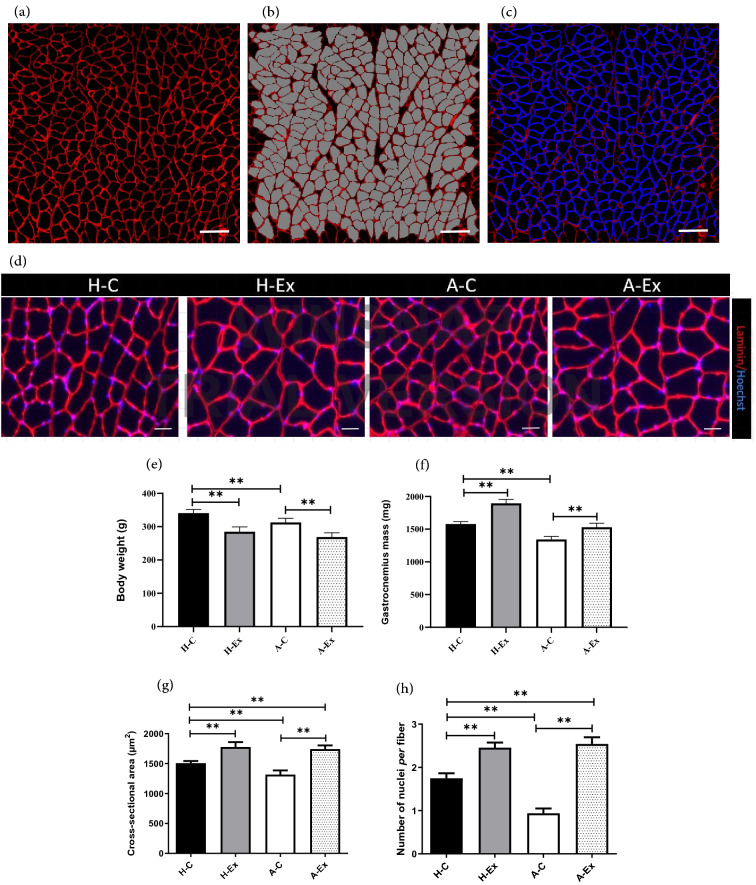
Muscle fiber cross-sectional area (CSA) in different experimental groups. (**a**) *Gastrocnemius* muscle cross section immunolabeled for Laminin (red). (**b**, **c**) Showing detection of region of interest and final detection process of myofibers by MyoView software. (**d**) Sections of gastrocnemius muscles immunolabeled for Laminin (red) and Hoechst (blue) for myonuclei detection process. (**e**) Body weight after training period. (**f**) *gastrocnemius* muscle weight. (**g**) Average *gastrocnemius* muscle CSA and (**h**) myonuclei numbers. Image analysis was performed using MyoView software^7^. Scale bar = 50 µm. **p < 0.001.

**Figure 4:**
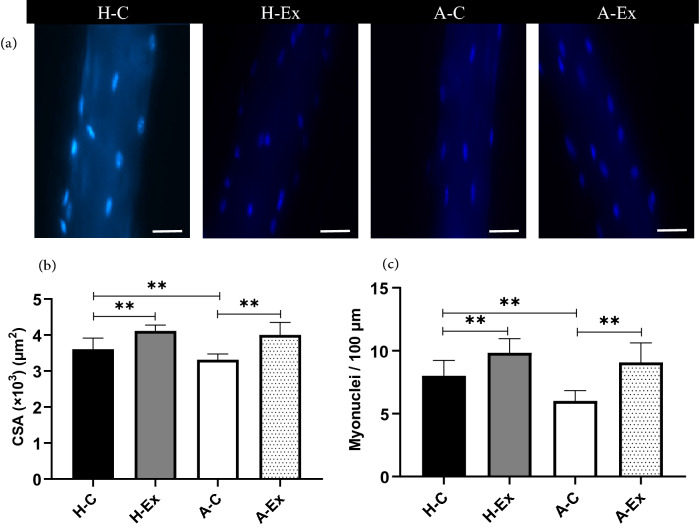
Morphometric analysis of myonuclei in *gastrocnemius* isolated myofibers in different experimental groups. (**a**) Representative image of nuclei stained by DAPI in isolated myofiber (Scale bar = 20 µm). (**b**) Quantification of CSA (× 10^3^) (µm^2^). (**c**) Number of myonuclei for 100 µm of isolated fibers. **p < 0.001.

After the training period, animals in A-C group had lower body weight and *gastrocnemius* muscle weight compared to the H-C group (*p* < 0.01; Fig. 3e, f). We assessed myonuclear number after the resistance training period in both hole muscle cross-section and isolated myofibers. The average muscle CSA in response to AD and resistance training is shown in Figs. 3 and 4. The CSA decreased significantly in the *gastrocnemius* skeletal muscle fibers of AD rats in A-C group compared to the H-C group (*p* < 0.001). In addition, compared to the H-C group, in the two groups that performed resistance training (H-Ex and A-Ex), mean CSA was significantly increased (*p* < 0.001).

The number of myonuclei in *gastrocnemius* skeletal muscle fibers followed the decrease of CSA in AD rats. The muscle nuclei count analysis showed that compared to the H-C group, the number of *gastrocnemius* muscle nuclei was significantly lower in A-C group (*p* < 0.01). Moreover, performing resistance training in H-Ex and A-Ex groups were resulted in an increase of the number of *gastrocnemius* myonuclei (*p* ≤ 0.001 and *p* ≤ 0.001, respectively).

### Resistance training effectively restored lower satellite cell content in AD myofibers

**Figure 5:**
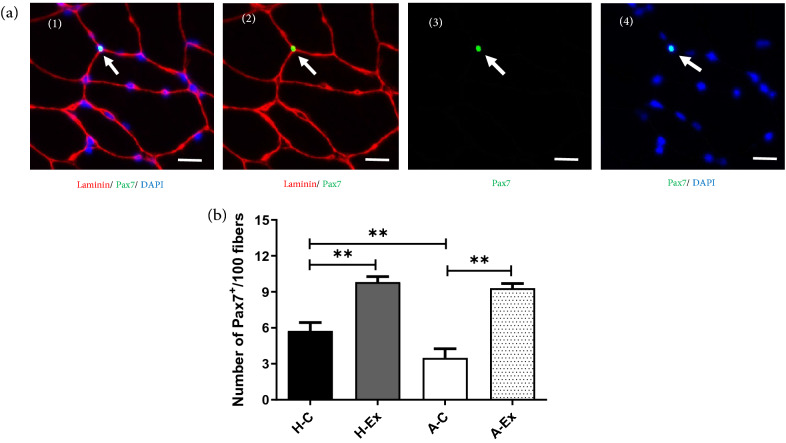
Muscle fiber satellite cell in different experimental groups. (**a** 1–4) Section of *gastrocnemius* muscle immunolabeled for Laminin (red), Pax7^+^ (green), and Hoechst (blue). (**b**) Number of Pax7^+^/100 fibers. Image analysis was performed using MyoView software^7^. Scale bar = 50 µm. **p < 0.001.

Further, we decided to quantify the number of satellite cells to determine the cause of the lower myonuclear number in *gastrocnemius* muscle of AD rats. To test this hypothesis, we further evaluated the number of Pax7^+^ cells in 100 fibers using MyoView software (Fig. 5). Alzheimer’s disease in A-C group was accompanied with decreased number of Pax7 positive cells in the *gastrocnemius* muscle compared to the H-C group (*p* < 0.001). Moreover, compared to the H-C group, the number of Pax7^+^ cells was significantly increased in H-Ex and A-Ex groups (*p* ≤ 0.001 and *p* ≤ 0.001, respectively).

### Resistance training increased the number of MyHC IIb fibers in AD myofibers

**Figure 6:**
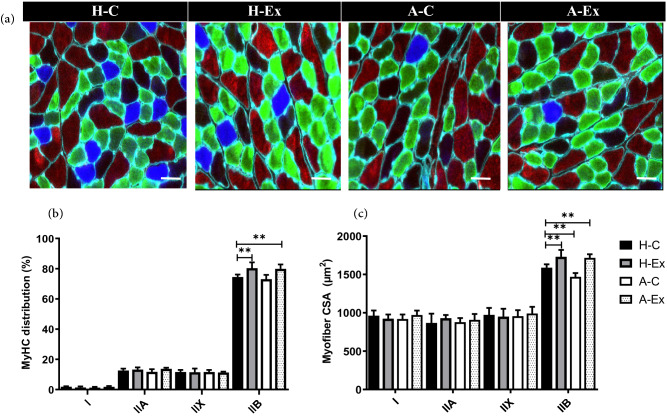
Muscle fiber myosin heavy chains in different experimental groups. (**a**) Sections of *gastrocnemius* muscle immunolabeled for MyHC I fibers (blue), MyHC IIa (green), MyHC IIx (black), and MyHC IIb (red). (**b**) The effect of resistance training on MyHC distribution (measured with MyoView). (**c**) The effect of resistance training on myofiber CSA in different fiber types (measured with MyoView). Image analysis was performed using MyoView software^7^. Scale bar = 50 µm. **p < 0.001.

We further evaluated the possible changes in the MyHC distribution and CSA in *gastrocnemius* muscle fibers from control and AD rats. The total number of fibers was similar in *gastrocnemius* muscle of the H-C (6582 ± 542), H-Ex (6748 ± 671), A-C (6462 ± 698), A-Ex (6651 ± 720) and there was no significant difference among the groups. There were no significant differences in the MyHC distributions of animals in A-C and H-C groups. However, resistance training significantly increased MyHC IIb distributions in animals from the H-Ex and A-Ex groups compared to their counterpart control groups (all *p* < 0.01). The mean value of MyHC I, IIa, and IIx fiber CSA were identical between all groups (all *p* > 0.05). However, MyHC IIb fiber CSA was smaller in animals from the A-C than in the H-C group (all *p* < 0.05). Resistance training significantly increased MyHC IIb fibers CSA in animals from the H-Ex and A-Ex groups compared to their counterpart control groups (all *p* < 0.01) (Fig. 6).

### Resistance training ameliorates oxidative stress in gastrocnemius muscle in an animal model of Alzheimer’s disease

**Figure 7:**
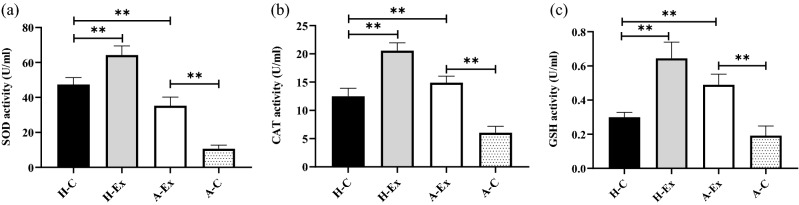
Resistance training ameliorates oxidative stress in *gastrocnemius* muscle in an animal model of Alzheimer’s disease. ELISA quantitative analysis of (**a**) Superoxide dismutase (SOD), (**b**) Catalase (CAT), and (**c**) glutathione peroxidase (GSH) in *gastrocnemius* muscle (Two-way ANOVA and subsequent Tukey’s HSD, *p* < 0.001).

Given that skeletal muscle growth is highly associated with oxidative stress, we investigated if resistance training plays a role in preventing oxidative stress in *gastrocnemius* muscle in an animal model of AD. As shown in Fig. 7, significant difference was observed in level of SOD, CAT, and GSH activities of different groups (all *p* < 0.001), indicating that SOD, CAT, and GSH activities of the *gastrocnemius* muscle in A-C rats were significantly lower than H-C rats (*p* < 0.001). Resistance training significantly increased SOD, CAT, and GSH activities in the *gastrocnemius* muscle of H-Ex and A-Ex groups compared to the H-C and A-C groups, respectively (*p* < 0.001). In general, these results suggest that AD could lead to impaired oxidative stress in the *gastrocnemius* muscle and resistance training played a positive role in improving this abnormality.

## Discussion

The current study examined whether AD and resistance training affected myonuclear number, satellite cell content, and MyHC distribution. Three novel results were obtained and discussed in more detail as follows: (1) AD causes a reduction in the myonuclear number and satellite cell content in AD myofibers, thereby causing muscle atrophy; (2) resistance training successfully restores AD-induced impairment in myonuclear number and satellite cell content thereby causing muscle hypertrophy; (3) resistance training-induced muscle hypertrophy is mediated by enhancement in MyHC IIb fiber CSA in AD myofibers.

Our results are consistent with previous studies^3,4^ that showed AD causes muscle atrophy, which was associated with a myonuclear reduction in AD rats. Although the exact mechanism of AD muscle atrophy is unclear, abnormal APP metabolism with amyloid-β deposition has been detected in skeletal muscle tissue of AD patients^5^. In this context, we were interested in knowing whether AD-induced muscle atrophy is related to myonuclear number. We observed that *gastrocnemius* muscle fiber CSA and myonuclear number were lower in the AD animals than in healthy rats. Moreover, it has been documented that fiber type distribution, may be linked to alterations in metabolic characteristics of chronic diseases^18^. In the present study, we found that *gastrocnemius* muscle in AD animals represents the same MyHC distribution compared with control animals. Although, MyHC IIb fiber CSA has decreased in AD myofibers indicating that this type of myofibers are more prone to atrophy in AD conditions. Although, our result showed that resistance training was effective enough to restore skeletal muscle fiber type CSA in AD animals. Previous studies suggest that reduced skeletal muscle strength is a risk factor for developing AD^19^ and lower muscle strength is also associated with greater cognitive impairment^3^. In addition, AD is associated with significant changes in body composition, including lean mass and bone density, suggesting that these changes are an early systemic manifestation of AD^4,20,21^.

Muscle satellite cell content was evaluated to understand how AD disrupts myonuclear number. Our results showed that AD decreases the number of Pax7^+^ cells in skeletal muscle of AD animals, suggesting AD suppresses muscle stem cell proliferation in skeletal muscle. It has been demonstrated that brain atrophy and muscle mass loss may co-occur in individuals with early AD and without cognitive impairment, suggesting the shared mechanisms between skeletal muscle atrophy and neuronal loss in the brain^4^. Since myonuclei are post-mitotic, it has been shown that satellite cell-mediated myonuclear accretion is required for skeletal muscle fiber hypertrophy^22^. In this regard, our results indicate that resistance training successfully restores satellite cell contents in AD myofibers. These findings suggest that skeletal muscle fiber atrophy in AD animals is related to the lower nuclear number, which might also be caused by failed nuclear accretion from satellite cells. In contrast, resistance training, thereby restoring satellite cell numbers, can produce significant physiological changes in hypertrophic stimuli. It has been demonstrated that skeletal muscle atrophy can elicit cognitive impairment in AD^23^. More importantly, it has been documented that skeletal muscle of AD animals are more prone to oxidative and inflammatory events^24^. In the present study we found that resistance training successfully restores AD-induced impairment in myonuclear number and satellite cell content, thereby causing muscle hypertrophy. Several studies have reported that physical exercise decreases the risk of onset and progression of AD^25,26^. Physical exercise also reduces age-related brain atrophy^27^, improves metabolic functions and mitochondrial biogenesis in the brain tissue^28^, reduces oxidative stress and neuroinflammation in the hippocampus^16^, and improves cerebral blood flow and cognitive impairments^29,30^. Although, the results of the present study should be interpreted regarding its limitations. First, we were unable to measure skeletal muscle strength to show the association between reduced muscle strength and the risk of developing AD. Second, we did not assess molecular signalling related to skeletal muscle atrophy and hypertrophy and future studies should focus on signalling pathways to understand the skeletal muscle plasticity in AD. Finally, we did not assess the characteristics of the brain tissue of AD animals in the present study.

Altogether, AD induces muscle atrophy and decrease myonuclear number and satellite cell content, while resistance training successfully restores these impairments. Thus, resistance training might serve as an adjunct therapy in AD. However, further work is necessary to further elucidate on the underlying mechanisms.

## Data Availability

The datasets used and/or analysed during the current study available from the corresponding author on resonable request.

## Abbreviations

AD: Alzheimer’s disease
SC: Satellite cell
MyHC: Myosin heavy chain
CSA: Cross-sectional area
Aβ_1–42_: Beta-amyloid 1–42
